# An optimized desuccinylase activity assay reveals a difference in desuccinylation activity between proliferative and differentiated cells

**DOI:** 10.1038/s41598-020-72833-7

**Published:** 2020-10-12

**Authors:** Taolin Yuan, Jaap Keijer, Angela H. Guo, David B. Lombard, Vincent C. J. de Boer

**Affiliations:** 1 Human and Animal Physiology, Wageningen University & Research, Wageningen, 6708 WD The Netherlands; 2 Department of Pathology, University of Michigan, Ann Arbor, MI 48109 USA

**Keywords:** Enzyme mechanisms, Enzyme mechanisms, Biochemical assays, Biochemical assays, Energy metabolism

## Abstract

Succinylation is a novel post-translational modification identified on many proteins and is involved in multiple biological processes. Succinylation levels are dynamically regulated, balanced by succinylation and desuccinylation processes, and are closely connected to metabolic state in vivo. Sirtuins have been shown to possess NAD^+^-dependent desuccinylation activity in vitro and in vivo, among which the desuccinylation activity of SIRT5 is most extensively studied. Our understanding of the response of succinylation levels to different metabolic conditions, is hampered by the lack of a fast NAD^+^-dependent desuccinylation assay in a physiological context. In the present study, we therefore optimized and validated a fluorescence-based assay for measuring NAD^+^-dependent desuccinylation activity in cell lysates. Our results demonstrated that shorter and stricter reaction time was critical to approach the initial rate of NAD^+^-dependent desuccinylation activity in crude cell lysate systems, as compared to the desuccinylation reaction of purified His-SIRT5. Analysis of desuccinylation activity in SIRT5 knockout HEK293T cells confirmed the relevance of SIRT5 in cellular desuccinylation activity, as well as the presence of other NAD^+^-dependent desuccinylase activities. In addition, we were able to analyse desuccinylation and deacetylation activity in multiple cell lines using this assay. We showed a remarkably higher desuccinylase activity, but not deacetylase activity, in proliferative cultured muscle and adipose cells in comparison with their differentiated counterparts. Our results reveal an alteration in NAD^+^-dependent desuccinylation activity under different metabolic states.

## Introduction

Proteins involved in almost all essential life processes undergo different post-translational modifications (PTMs), by which protein structural changes are introduced and the functionality of the proteome is diversified^1–3^. Post-translational modifications are closely associated with metabolites^4–6^, and the intimate connection between PTMs and metabolites allow PTMs to integrate different signals to enable rapid responses of the organism to environmental challenges^7^. Lysine succinylation is a relatively new PTM, identified nearly one decade ago^1^. It provides a dynamic and relatively abundant network of modifications on both mitochondrial and extra-mitochondrial proteins^1,8–10^. Lysine residues of many proteins involved in important metabolic processes, such as fatty acid β-oxidation, ketogenesis, tricarboxylic acid (TCA) cycle, branched chain amino acids metabolism, and glycolysis/gluconeogenesis, are subjected to succinylation^11–16^. In addition, protein succinylation levels respond to metabolic manipulations. For example, succinylation levels in whole-cell extracts and mitochondria in mouse liver were increased under a fasting condition^11^. In contrast, mouse liver succinylation levels were shown to be significantly decreased^17^ or variably altered^18^ by a high fat diet intervention. These results indicate that protein succinylation levels vary with metabolic contexts and are likely to play a role in various signaling processes and regulatory pathways. Therefore, succinylation levels need to be carefully controlled. Sirtuins, a family of nicotinamide adenine dinucleotide (NAD^+^)-dependent lysine deacylase, regulate acylation levels in vivo^19–23^. Thus far, sirtuin (SIRT) 5 and SIRT7 have been shown to be able to remove a succinyl adduct from lysine in the presence of the cofactor NAD^+^^24,25^. SIRT5 was identified as a robust desuccinylase^24^, but it can also remove malonyl and glutaryl adducts from lysine residues with high affinity^24,26^ and possesses a weak deacetylase activity^27^. SIRT7 was demonstrated to function as a histone desuccinylase^25^, and is also able to remove acetyl-group from targets^28,29^. Since the protein succinylation level can be driven by succinyl-CoA levels^5,8,30^, as well as NAD^+^ levels or the NAD^+^/NADH balance^31^, it is likely that ultimately a complex interplay between succinyl-CoA, NAD^+^ and desuccinylase activity dictates lysine succinylation levels^15,16^. Although changes in succinylation levels have been observed with altered metabolism, it is not completely understood how desuccinylation activity responds to metabolic alterations. Improved understanding would be facilitated by the availability of additional options to assay desuccinylase activity.

A fluorescence based, homogenous, NAD^+^-dependent desuccinylation activity assay to study desuccinylase activity in crude cell lysate directly and rapidly is lacking. Its availability would allow for studying desuccinylation in a physiological context. Fluorogenic assays have been used for identifying activators and inhibitors of recombinant SIRT5 in vitro^32–34^. Only a limited number of studies analysed desuccinylase activity in the more physiological context of a cell lysate^35,36^. Notably, NAD^+^-dependent desuccinylase activity in a crude cellular extract has not yet been reported to our knowledge. Here, we optimize and validate a fluorescence-based assay for detecting NAD^+^-dependent cellular desuccinylase activity. Moreover, we use this assay to gain insights into NAD^+^-dependent desuccinylase activity in different cellular contexts, as well as its relation to global protein succinylation levels.

## Results

### Optimization and validation of desuccinylation activity in crude cell lysate

**Figure 1:**
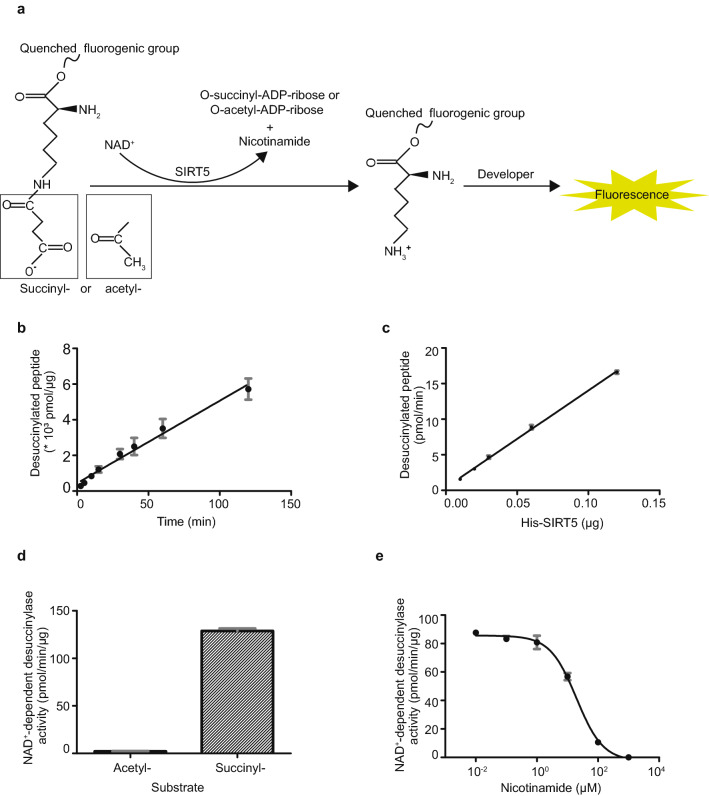
NAD^+^-dependent deacylation activity of human His-SIRT5. (**a**) Schematic illustration of desuccinylation and deacetylation activity analysis using a fluorogenic reporter assay. (**b**) Time course NAD^+^-dependent desuccinylation of succinylated-substrate by His-SIRT5 (0.046 µg). The desuccinylation reaction was proceeded for 2.5–120 min. Data represent mean ± SEM, n = 3. (**c**) NAD^+^-dependent desuccinylation of succinylated-substrate by increasing His-SIRT5 input (0.03–0.12 µg). (**d**) NAD^+^-dependent deacetylation (left) and desuccinylation activity (right) of His-SIRT5 (0.03 µg). (**e**) Inhibition of NAD^+^-dependent desuccinylation activity of His-SIRT5 (0.06 µg) by nicotinamide. (**b**–**e**), fluorescence signal obtained in the negative group (with cell lysates and with acylated-substrate and without NAD^+^) was subtracted from each experiment. Error bars in (**c**–**e**) represent deviation from the mean of duplicates.

**Figure 2:**
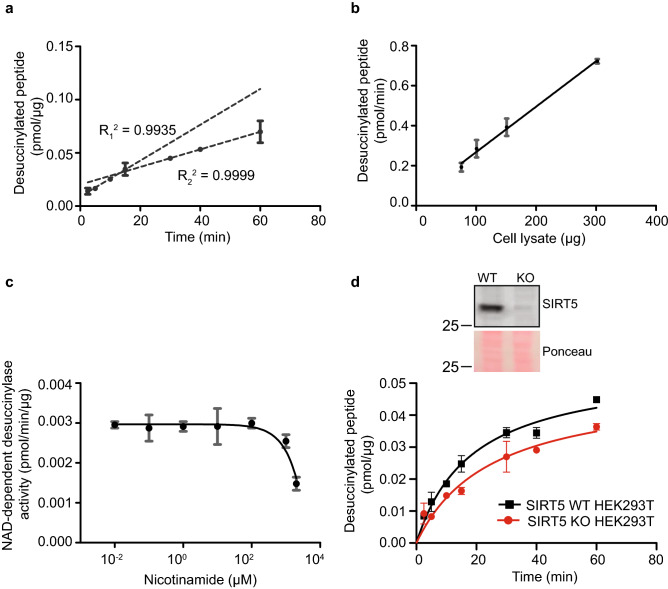
NAD^+^-dependent desuccinylation activity in human fibroblast lysate. (**a**) Time course NAD^+^-dependent desuccinylation of succinylated-substrate by cell lysate (148.5 µg). The desuccinylation reaction was proceeded for 2.5–60 min. Two dashed lines represent two different slopes obtained from the first four data points and last three data points, respectively. (**b**) NAD^+^-dependent desuccinylation of succinylated-substrate by increasing fibroblasts lysate (75.5–302 µg). (**c**) Inhibition of NAD^+^-dependent desuccinylation activity of fibroblasts lysate (187.3 µg) by nicotinamide. (**d**) Time-course NAD^+^-dependent desuccinylation of succinylated-substrate by SIRT5 WT (140 µg) and SIRT5 KO (140 µg) HEK293T cell lysate, respectively. Blots of SIRT5 and ponceau staining were cropped from the same blot, and the full-length SIRT5 blot and the ponceau image are presented in Supplementary Fig. 2. (**a**–**d**) Fluorescence signal obtained in the presence of substrate and absence of NAD^+^ was subtracted from each experiment sample. Experiments were done in duplicates, and error bars represent deviation from the mean.

We aimed to measure cellular NAD^+^-dependent desuccinylase activity by using the succinylated-fluorogenic reporter substrate (Fig. 1a). Given the complexity of enzymatic reactions in crude cell lysates, we first optimized and validated the assay with a pure recombinant NAD^+^-dependent desuccinylase protein, His-SIRT5. Levels of desuccinylated peptides increased linearly over time up to 2 h (Fig. 1b), when incubating His-SIRT5 with 10 µM succinyl-substrate and 500 µM NAD^+^. In addition, the desuccinylation rate of the peptide substrate linearly increased with the His-SIRT5 input when the reaction time was fixed (Fig. 1c). In contrast to the substantial desuccinylation activity of His-SIRT5, its deacetylation activity was virtually undetectable (Fig. 1d). In addition, we performed the assay in the presence of nicotinamide (NAM), a well-known inhibitor of sirtuins. As expected, the desuccinylation activity of His-SIRT5 was negatively impacted by NAM, with an IC_50_ value of 20.2 µM (Fig. 1e). These results showed that NAD^+^-dependent desuccinylation activity of His-SIRT5 can be analysed well using the succinylated-fluorogenic reporter substrate. We next examined whether the assay set-up for pure His-SIRT5 could be applied to measure desuccinylase activity in a crude cell lysate. Linearity of cellular desuccinylation activity with time was assessed in fibroblasts cell lysates. Desuccinylation of the succinylated-substrate was proportional to reaction time up to 15 min, after which the reaction rate decreased (Fig. 2a), suggesting that an initial reaction rate was reached in the first 15 min. After 15 min, desuccinylated adduct amount again increased linearly with time, but at a slower reaction rate. Based on this, we performed further analyses of desuccinylase activity after 10 min, i.e. within the first 15 min. Desuccinylation by cell lysate after 10 min reaction time increased linearly in a protein-dependent manner (Fig. 2b). Inhibition by NAM of the cellular desuccinylation activity was observed at concentrations higher than 0.1 mM, and maximal inhibition was seen at 2 mM (Fig. 2c). Moreover, the desuccinylase activity was lower in HEK293T SIRT5 knockout cell lysates as compared to wild type cell lysates at all tested reaction time, which ranged from 2.5 to 60 min (Fig. 2d). This result demonstrates that SIRT5 contributes to the desuccinylation activity of the crude cell lysate. Of note, the level of desuccinylated adduct increased over time in the absence of SIRT5 (Fig. 2d), indicating the presence of multiple desuccinylation enzymes in the crude cell lysate. Taken together, our results show that cellular desuccinylation activity can be analysed by the fluorescence-based assay, is dependent on NAD^+^ and is sensitive to NAM inhibition.

### Desuccinylation and deacetylation activities in a range of cells with diverse metabolic states

**Figure 3:**
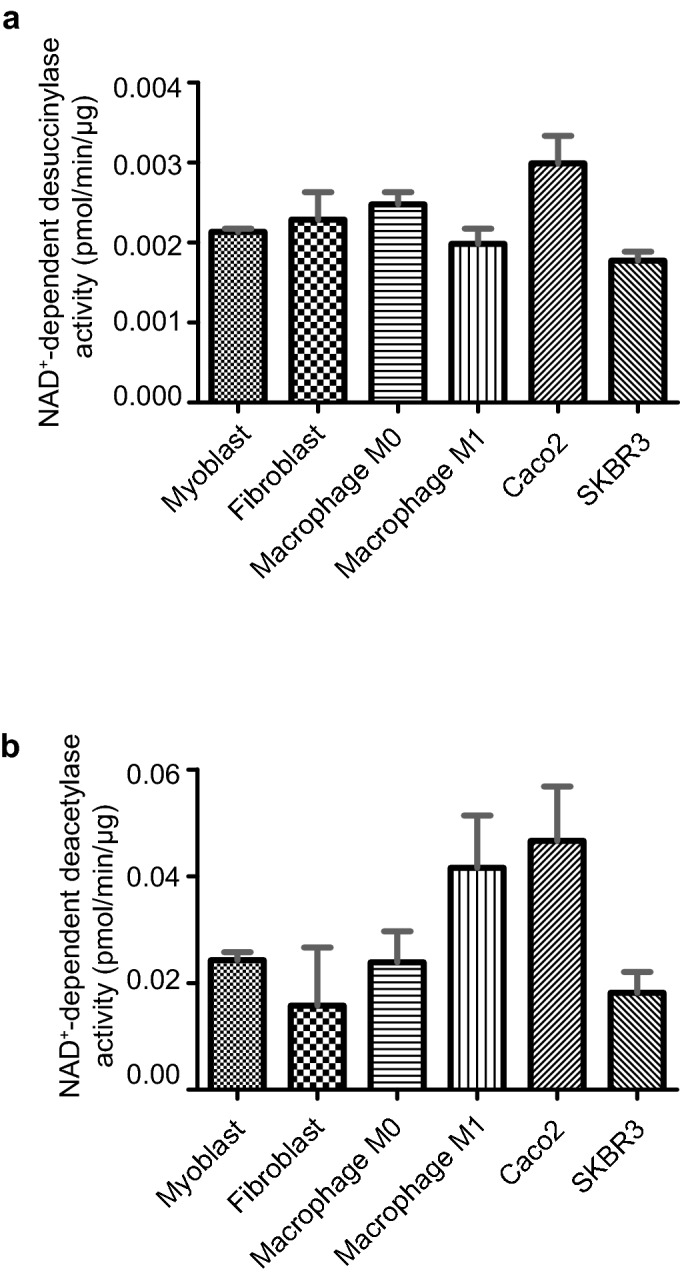
NAD^+^-dependent deacylation activities in various cell lines. (**a**) NAD^+^-dependent desuccinylase activity, and (**b**) NAD^+^-dependent deacetylase activity in multiple cell lines. 200 µg of cell lysates, 10 µM of succinyl/acetyl-substrate and 500 µM NAD^+^ were present in the desuccinylation/deacetylation assays. Fluorescence signal obtained in the presence of substrate and absence of NAD^+^ was subtracted from each experiment sample. Experiments were done in duplicates, and error bars represent deviation from the mean.

Cellular succinylation levels were shown to be dynamically affected by metabolism^8^, whereas little is known about NAD^+^-dependent desuccinylation activity in cells with variable metabolic states. To gain insights into the latter, we analysed the desuccinylation activity by applying the fluorescence-based assay in a variety of cell lines that are commonly used. Desuccinylation activities among the different cell lines ranged from 0.0018 to 0.003 pmol/min/µg protein (Fig. 3a), whereas the deacetylation activities in the same cell lines were approximately tenfold higher (Fig. 3b). Interestingly, desuccinylation activity among the tested cell lines did not differ markedly, whereas differences in the deacetylase activity appeared to larger between the cell types. The deacetylation activity was highest in Caco2 cells and lowest in fibroblasts (Fig. 3b).

### A difference in desuccinylation activity between proliferative and differentiated cells

**Figure 4:**
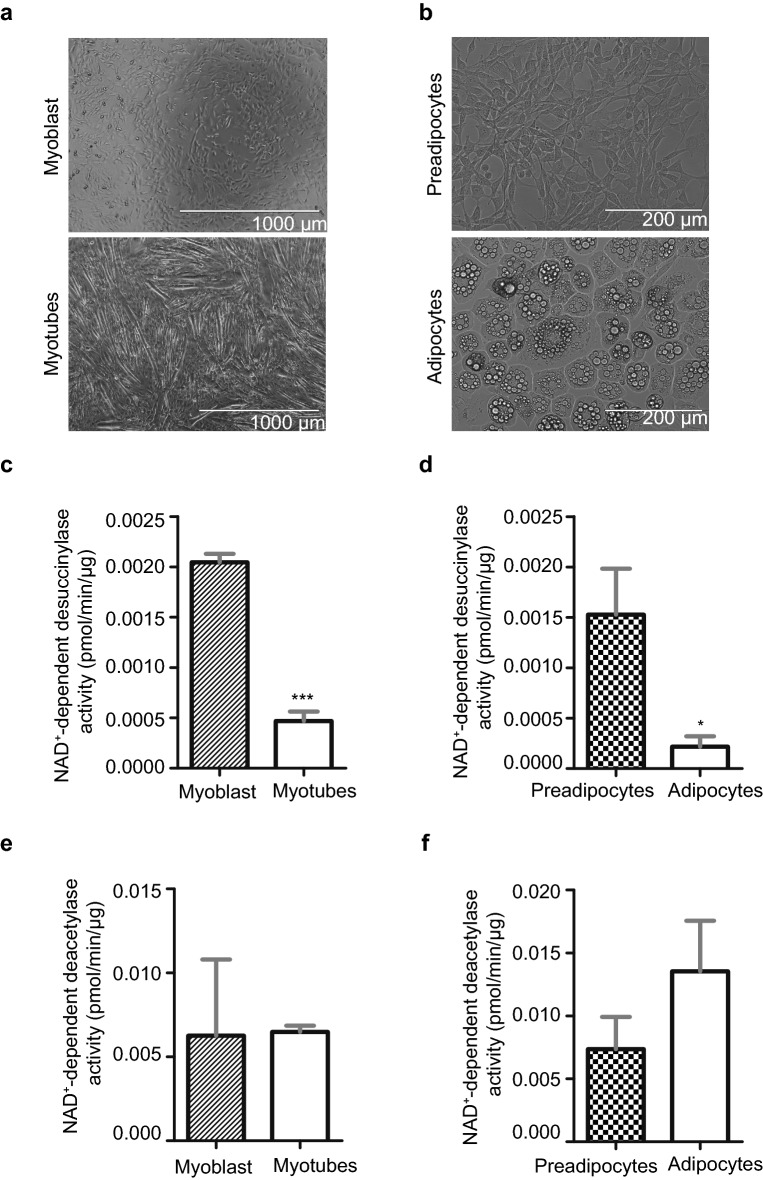
NAD^+^-dependent deacylase activities in C2C12 and 3T3-L1 cell lysates. (**a**) Light micrograph of myoblasts (left panel), and myotubes at day 6 of differentiation (right panel); scale bar is 1000 µm. (**b**) Light micrograph of preadipocytes (left panel), and adipocytes at day 11 of differentiation (right panel); scale bar is 200 µm. NAD^+^-dependent desuccinylase activity in 150 µg lysates of (**c**) myoblast and myotubes and (**d**) 180 µg lysates of preadipocytes and adipocytes. NAD^+^-dependent deacetylase activity in lysates (145.6 µg) of (**e**) myoblasts and myotubes and (**f**) in lysates (145.6 µg) of preadipocytes and adipocytes. Date represent mean ± SEM. n = 3 for desuccinylation activity assays. Two-tailed unpaired Student’s t-test was performed. *P < 0.05, ***P < 0.001. n = 2 for deacetylation activity assays.

**Figure 5:**
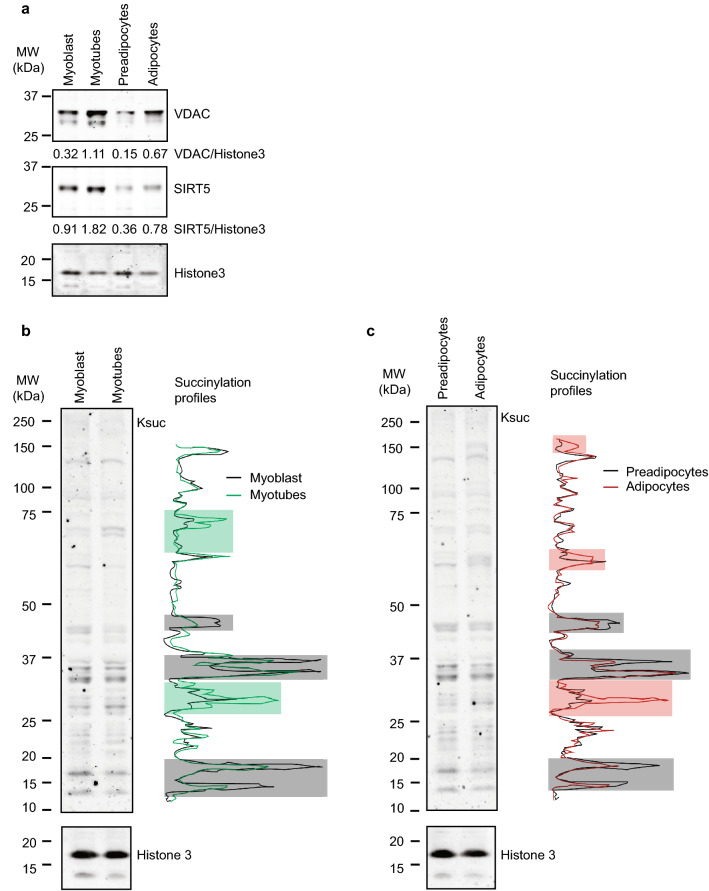
SIRT5 protein and succinylation levels in C2C12 myoblasts and myotubes and in 3T3-L1 preadipocytes and adipocytes. (**a**) Western blot analysis of voltage-dependent anion channel (VDAC), SIRT5 and histone 3. The numbers represent band intensity relative to histone 3. Blots of VDAC and SIRT5 were cropped from the same gel, and histone 3 was cropped from a different gel. Full-length blots are presented in Supplementary Fig. 3. (**b**) Succinyllysine levels and blot lane profiles of C2C12 myoblasts (black line) and myotubes (green line). Grey areas represent succinyllysine protein bands with higher intensity in myoblasts and green areas represent succinyllysine protein bands with lower intensity in myoblasts. (**c**) Succinyllysine levels and blot lane profiles of 3T3-L1 preadipocytes (black line) and adipocytes (red line). Grey areas represent succinyllysine protein bands with higher intensity in preadipocytes and red areas represent succinyllysine protein bands with lower intensity in adipocytes. In (**b**,**c**), blots of succinylation and histone3 were grouped from different gels, and full-length blots for (**b**,**c**) are presented in Supplementary Fig. 4.

Next, we asked whether cellular differentiation alters desuccinylase activity. In myoblasts differentiated into myotubes, metabolic changes occur with a relative increase in mitochondrial mass^37,38^. Furthermore, differentiation of preadipocytes to adipocytes results in lipid droplets formation and a relative increase in mitochondrial biogenesis and metabolism^39,40^. Since SIRT5 is mainly localized in mitochondria, and acylations such as acetylation has been shown to be involved in cell differentiation^41,42^, desuccinylase activity could play a role in differentiation as well. C2C12 mouse myoblasts were successfully differentiated into myotubes, as can be seen from the longer myotubular structures that were observed after differentiation (Fig. 4a). Also, 3T3-L1 mouse preadipocytes were successfully differentiated into adipocytes, as can be seen from the lipid droplet accumulation in the cytosol of the adipocytes (Fig. 4b). Interestingly, desuccinylation activity in myotubes was around fourfold (P = 0.0002) lower than in the myoblasts (Fig. 4c) and desuccinylase activity in adipocytes was around sixfold (P = 0.0375) lower than that in the preadipocytes (Fig. 4d). In contrast, NAD^+^-dependent deacetylase activity displayed comparable in myotubes and adipocytes as compared to that in myoblast and preadipocytes, respectively (Fig. 4e,f). Given that the observed NAD^+^-dependent desuccinylation activities in myotubes and adipocytes were much lower than that in their proliferative counterparts, we aimed to study whether this was reflected in the SIRT5 protein levels. Since mitochondrial biogenesis is closely associated with cell differentiation, and SIRT5 is known to be the non-redundant desuccinylase in mitochondria. We first analysed the expression level of the mitochondrial outer membrane protein, voltage-dependent anion channel (VDAC). As expected, protein levels of VDAC (normalised to histone3 level) were increased by around fourfold in both myotubes and adipocytes (Fig. 5a). Unexpectedly, the SIRT5 protein levels (normalised to histone3 level) in myotubes and adipocytes were increased by twofold as compared to their proliferative counterparts (Fig. 5a). Since total desuccinylase activity decreased (Fig. 4c,d) and SIRT5 protein levels increased, we were interested to see how it was reflected in the protein lysine succinylation levels of undifferentiated and differentiated myocytes and adipocytes. Our results show a distinct pattern of lysine succinylation for each cell type (Fig. 5b,c). Band intensity lane profiling of succinylated proteins on blots showed multiple bands with a higher intensity in the myoblasts compared to myotubes (grey areas in Fig. 5b), indicating that higher lysine succinylation levels of these proteins in myoblasts. However, other protein bands were of lower intensity in the myoblasts as compared to the same bands in the myotubes (green areas, Fig. 5b), indicating that lower succinylation levels of these proteins in myoblasts as compared to myotubes. Also when comparing succinylation profiles between preadipocytes and adipocytes, succinylation differences similar to those seen in the muscle cell were observed, with multiple protein bands being of higher intensity in preadipocytes as compared to adipocytes (grey areas, Fig. 5c), while this was the other way around for other proteins (red areas, Fig. 5c). Taken together, in differentiated myotubes and adipocytes, the overall desuccinylation activity was decreased, the SIRT5 protein level was increased and protein lysine succinylation profiles changed differentially.

## Discussion

The complex interplay between metabolite-driven succinylation and NAD^+^-dependent desuccinylation gives opportunities to cells to regulate protein function based on metabolic state of the cells. To get more insights into these processes, we optimized a desuccinylase assay in cell lysates and applied our method to different cell types and conditions. We showed that a stricter reaction time was required to analyse initial rate in crude cell lysates as compared to recombinant His-SIRT5, that SIRT5 was responsible partially for the observed NAD^+^-dependent desuccinylase activity, and that NAD^+^-dependent desuccinylation activity varied with physiologic states. NAD^+^-dependent desuccinylase activity was higher in proliferative myoblasts and preadipocytes as compared to their differentiated counterparts.

Protein acetylation has been demonstrated to act as a regulator of cell differentiation from myoblast to myotubes^41^ as well as from preadipocyte to adipocytes^42^. Acetylation of histones is regulated by deacetylases and acetyltransferase to maintain, activate or repress gene transcription programs^43–45^. Although succinylation has been identified on histones, and NAD^+^-dependent desuccinylation by SIRT7 of specific histone marks regulated DNA damage repair pathway^25^, it is not known whether there is a general role for succinylation and desuccinylation in regulating cell differentiation. Our findings that NAD^+^-dependent desuccinylase activities are higher in proliferative cells as compared to their differentiated counterparts, suggest that desuccinylation could play a role in the differentiation process. Interestingly, the NAD^+^-dependent desuccinylase protein, SIRT5, has been shown to support proliferative tumor cell growth in in vitro as well as in vivo models^46–48^. SIRT5 has also been found to be upregulated in different tumor types and its increased expression was proposed as a marker for recurrence in non-small cell lung cancer (NSCLC)^49,50^.

In our study, we identified a mismatch between SIRT5 protein expression level and NAD^+^-dependent desuccinylase activity. SIRT5 protein was expressed higher in differentiated cells as compared to proliferative cells, whereas desuccinylase activity was lower. The increased SIRT5 protein level in differentiated myotubes and adipocytes, could be explained by the fact that SIRT5 is primarily located in mitochondria^11^, and that increased mitochondrial biogenesis is associated with both myogenesis^37^ as well as adipogenesis^39^. In support of this, protein levels of VDAC, an abundant mitochondrial outer membrane protein, were increased in differentiated cells as compared to proliferative cells. Interestingly, that the increased protein expression of SIRT5 did not translate into a higher NAD^+^-dependent desuccinylase activity could implicate that mitochondria-localized SIRT5 is post-translationally modified to be inactive. Since we analysed enzymatic activity in cell lysates and added external NAD^+^, NAD^+^ could not be a limitation for the analysed desuccinylase activity. Instead, SIRT5 could be regulated by PTMs or could be inhibited by interaction with other proteins that are part of a SIRT5 regulatory complex. Although only a limited number of SIRT5 protein interactors in the mitochondria have been identified in a large screen for mitochondrial sirtuin interactions^51^, it is possible that these interactors could have a role in altering the desuccinylation activity of SIRT5. For SIRT1, post-translational modifications and their impacts have been studied extensively. For example, phosphorylation on several SIRT1 sites could translocate SIRT1 and redirect it to specific targets with enhanced its enzymatic function^52,53^. Additionally, other PTMs have also been demonstrated to affect SIRT1 level or activity, including sumoylation^54^, carbonylation^55^, and methylation^56^. Another explanation for the mismatch between the SIRT5 expression and the desuccinylase activity could be that mitochondrial SIRT5 is not the main contributor to overall NAD^+^-dependent desuccinylase activity in myoblasts and preadipocytes. Indeed, when analysing NAD^+^-dependent desuccinylase activity in cell lysates of HEK293T SIRT5 KO cells, we observed substantial NAD^+^-dependent desuccinylase activity, whereas SIRT5 protein was not detectable. These findings highlight the importance of analysing NAD^+^-dependent desuccinylase activity, over only analysing expression of the protein or gene.

It has become apparent that sirtuin proteins have versatile deacylase activity^21,24,57^. Sirtuin members share deacylase activities among each other, but also specific deacylase, ADP-ribosyltransferase^58,59^ and lipoamidase^60^ activities have been identified for sirtuins. Deacetylation activity is shared by SIRT1^19^, SIRT2^22^, and SIRT3^20^. Desuccinylation has been identified, apart from SIRT5 and SIRT7, for SIRT3 and SIRT4 although these sirtuins only displayed minor desuccinylation activity against a succinylated lysine peptide in vitro^61^.

The versatility in enzymatic activity of sirtuins is also apparent from studies on SIRT6, which has both deacetylase activity as well as de-fatty acylase activity and ADP-ribosyltransferase activity^59,62,63^. Specific mutations of SIRT6 identified in tumors could even predispose for one activity over the other^64^. In our cell lines and physiological conditions tested, the NAD^+^-dependent desuccinylase activities of cell lysates were approximately tenfold lower than the NAD^+^-dependent deacetylase activities. Interestingly, the deacetylase activity did not change upon differentiation, whereas desuccinylase activity was different. Interestingly, in our study, we even identified two distinct desuccinylation reaction rates in cell lysates, one initial high activity and a second slower one. This could possibly be explained by differential substrate-binding affinity by multiple desuccinylases. Further studies are needed to explore the desuccinylation potential of specific proteins.

Overall, we optimized a fluorescence-based assay for detecting cellular NAD^+^-dependent desuccinylase activity, and demonstrated that NAD^+^-dependent desuccinylase activity is lowered in differentiated myotubes and adipocytes. The observed mismatch between SIRT5 protein expression level and NAD^+^-dependent desuccinylase activity highlights the relevance of analysing sirtuin enzymatic activities in physiological contexts. The high desuccinylase activity in proliferating cells is of relevance to analyse how desuccinylase activity contributes to tumor cell function and how sirtuins with NAD^+^-dependent desuccinylase activity are regulated during proliferation and differentiation.

## Material and methods

### His-SIRT5 expression and purification

The human His-SIRT5 (6 × His) plasmid was a gift from Cheryl Arrowsmith (Addgene plasmid # 25487). Human SIRT5 gene is inserted into the vector pET28a-LIC, and six histidine residues are fused into the SIRT5 protein on N terminal, and the plasmid is resistant to kanamycin. The plasmid was transformed into chemical competent *E. coli* BL21 by heat shock as previously published^65^, with minor modifications. Briefly, 1 µg of plasmid DNA was added to 50 µl competent *E. coli* BL21 cells, incubated for 30 min on ice, heat-shocked for 11 s at 42 °C and returned to ice for 5 min. After the cells were incubated in 950 µl Lysogeny broth (LB) medium for 1 h at 37 °C. 100 µl of the transformation mix was plated on LB agar plates containing 50 µg/ml kanamycin and incubated overnight at 37 °C. For His-SIRT5 protein expression level, a single colony was inoculated in 3 ml LB medium with 50 µg/ml kanamycin, incubated for 8 h at 37 °C with shaking at 220 rpm using an incubator shaker (G25, New Brunswick Scientific), and the bacterial starter culture was stored at 4 °C. Next day, 100 µl of bacterial starter culture was inoculated to 100 ml LB medium with 50 µg/ml kanamycin in a 300 ml Erlenmeyer flask, incubated at 37 °C with shaking at 220 rpm until an OD_600_ of 0.25, after which the cultures were placed at 22 °C and Isopropyl β-d-1-thiogalactopyranoside (IPTG) was added to a final concentration of 1 mM. Cells were harvested 18 h after IPTG induction by centrifugation at 6000 × *g* for 15 min at 4 °C. For purification of His-SIRT5, 3–4 mg of *E.coli* pellet was resuspended in 3 ml lysis buffer (10 mM Tris/HCl pH 8.0, 500 mM NaCl, and 1 mg/ml lysozyme) and incubated end-over-end for 30 min at 4 °C. Cells were then sonicated at 40% amplitude (burst of 1 s followed by 3 s interval) for 5 min on ice using a digital sonifier (Branson SLPe). Insoluble debris in the cell lysate was removed by centrifugation at 8500 × *g* and 4 °C for 15 min. Imidazole/HCl at pH 8.0 was added to the supernatant to a final concentration of 30 mM. Crude His-SIRT5 was purified using HisLink protein purification resin (#V8823, Promega) in combination with a chromatography column (#731-1150, Bio-Rad). The protein purification resin consists of microporous silica resin which contains a high level of tetradentate-chelated nickel and can purify polyhistidine-tagged recombinant proteins efficiently. To purify crude His-SIRT5, 0.4 ml of the HisLink protein purification resin was loaded to a chromatography column, then the resin was first equilibrated by washing five times with washing buffer (25 mM Tris/HCl pH 8.0, 500 mM NaCl, and 30 mM imidazole). *E. coli* lysate was then loaded onto the equilibrated column and was allowed to pass through, and this step was repeated once. Then, the column was washed five times with the washing buffer, and His-SIRT5 was eluted with 200 µl of elution buffer (25 mM Tris/HCl pH 8.0, 100 mM NaCl, and 500 mM imidazole). Elution fractions were checked for SIRT5 content using gel electrophoresis and Coomassie staining (Supplementary Fig. 1). Prior to use in further experiments, imidazole was removed from the eluted His-SIRT5 using ultra-0.5 centrifugal filter units (#UFC501096, Merck Millipore) according to the manufacturer’s instruction. Briefly, 50 µl of eluted His-SIRT5 was loaded to a filter column, and 450 µl of exchange buffer (25 mM Tris/HCl pH 8.0 and 100 mM NaCl) was added, followed by centrifugation at 14,000 × *g* for 15 min at 4 °C. The flow through was discarded and another 450 µl of exchange buffer was added to the column and the same centrifugation step was repeated once. Then the His-SIRT5 was collected by reverse centrifugation in an Eppendorf tube at 1000 × *g* for 2 min at 4 °C. The protein concentration of His-SIRT5 was determined using a Bradford protein assay (#23200, Thermo Scientific), after which the His-SIRT5 was preserved in 1 × freezing buffer (50 mM Tris/HCl pH 8.0, 500 mM NaCl, 500 mM DTT, and 20% glycerol) and aliquoted with 10 µl/tube and the aliquots were stored at − 80 °C until use.

### Cell culture

Murine 3T3-L1 preadipocytes (ATCC, #CL-173) were cultured at 37 °C, 5% CO_2_ in 3T3-L1 medium, which consisted of DMEM (#11880-028, Gibco) supplemented with 10% (v/v) foetal bovine serum (FBS, #06Q3501K, Gibco), 4.5 g/l glucose (#G7021, Sigma-Aldrich), 4 mM l-glutamine (#25030-024, Gibco) and an antibiotic–antimycotic mix (10,000 units/ml of penicillin, 10,000 µg/ml of streptomycin, and 25 µg/ml Amphotericin B, #15240-062, Gibco). For differentiation of preadipocytes into adipocytes, 3T3-L1 preadipocytes (8,000 cells/well) were first seeded in 6-well plates in 3T3-L1 medium, and differentiation was started 48 h after the preadipocytes reached 100% confluency by switching to 3T3-L1 differentiation medium (2 ml/well) in the same culture plates. The 3T3-L1 differentiation medium consisted of 3T3-L1 medium supplemented with a final concentration of 1 µM insulin (#I0516, Sigma-Aldrich), 0.5 mM 3-isobutyl-1-methylxanthine (#5879, Sigma-Aldrich), 1 µM dexamethasone (#D4902, Sigma-Aldrich) and 1 µM 15-Deoxy-Δ12,14-prostaglandin J2 (#D8440, Sigma-Aldrich). The cells were cultured in the differentiation medium for 48 h, then in differentiation maintaining medium (3T3-L1 medium containing 1 µM insulin) until the cells were harvested at day 11 of differentiation.

Murine C2C12 myoblasts (ATCC, #CRL-1772) were cultured in C2C12 growth medium, consisted of DMEM (#11960-044, Gibco) supplemented with 25 mM HEPES (#15630080, Thermo Scientific), 10% FBS, and antibiotic–antimycotic mix. For differentiation of myoblasts into myotubes, myoblasts (3 × 10^5^ cells/well) were first seeded in a 6-well plate in the C2C12 growth medium, and differentiation of myoblasts was started when the myoblasts reached 90% confluency by switching to C2C12 differentiation medium that contained 2% horse serum (#26050070, Gibco) instead of 10% FBS. Cells were cultured in the C2C12 differentiation medium until harvest at day 6 of differentiation.

### Generation of SIRT5 knockout HEK293T cells

SIRT5 knockout HEK293T cells were generated using the CRISPR-Cas9 system following the directions outline in Ran et al.^66^. Briefly, sgRNA against SIRT5 oligos (sequence: GGCTGCTGGGTACACCACAG) were resuspended in water, annealed, then phosphorylated using T4 polynucleotide kinase. Then, the ligated oligos and PX459 vector were digested and ligated together using BbsI and the T7 ligase, respectively. HEK293T cells (ATCC, #CRL-11268), cultured following ATCC guidelines, were transfected with the PX459-sgRNA vector using Lipofectamine 3000 according to manufacturer’s instructions. Transfected cells were selected using puromycin (3 µg/ml) for 48 h. To achieve single-cell colonies, cells remaining after selection were diluted to a density of 50 cells/10 ml of media, and 100 µl of the diluted cell suspension per well was plated to 96-well plates. SIRT5 status in the colonies were assessed using immunoblotting. Colonies lacking SIRT5 protein were then sequenced to confirm Cas9-mediated genomic alterations at the location targeted by the sgRNA. Colonies with SIRT5 levels comparable to WT HEK293T cells were used as controls for SIRT5 KO cells.

### Desuccinylation activity assays

Desuccinylation activity was measured using a fluorogenic reporter assay. Succinylated-substrate (#BML-KI590), assay buffer (#BML-KI286-0020), developer concentrate (20x, #BML-KI105), NAD^+^ (#BML-KI282), and NAM (#BML-KI283-0500) were all purchased from Enzo. Assay reagents were prepared freshly in the assay buffer prior to the assay and were kept on ice until use.

For desuccinylase activity in cells, trypsinized, washed and pelleted cells were resuspended in lysis buffer (50 mM Tris/HCl, pH 8.0,137 mM NaCl, 2.7 mM KCl, 1 mM MgCl_2_, 0.1% Triton X-100, and protease inhibitor (#04693159001, Roche)) and sonicated on ice at 40% amplitude (burst of 1 s followed by 3 s cooling) for 12 bursts using a Branson SLPe digital sonifier. Cell lysates were centrifuged at 10,000 × *g*, 4 °C for 10 min, and the supernatants were used in further experiments. Samples were equalized with lysis buffer on protein concentration after determination using DC protein assays (#5000116, Bio-Rad). Protein input for each sample in the desuccinylase reaction was indicated in the figure legends. Prior to use in desuccinylation reaction, 28 µl of cell lysates were incubated at a 37 °C water bath with shaking at 60 rpm (Shaking water bath, VWR) in 1.5 ml Eppendorf tubes for 10 min to degrade endogenous NAD^+^, then 0.6 µl of 50 mM dithiothreitol^67^ dissolved in assay buffer was added to all cell lysates, and incubation continued for an additional 5 min. The catalytic reactions were performed at 37 °C with shaking at 60 rpm, initiated by adding 28 µl of pre-warmed (37 °C) 20 µM succinyl-substrate (final concentration of 10 µM) in combination with either 1 mM NAD^+^ (final concentration of 500 µM) or assay buffer only (negative control). After adding the substrates, the tubes were vortexed at 2200 rpm for 2 s with a Minishaker (IKA MS2) and then placed at the 37 °C water bath. The reactions were stopped after 10 min, unless stated otherwise, by adding 56 µl of 1 × developer containing 2 mM NAM, vortexing at 2200 rpm for 2 s, then incubating at 25 °C for 15 min. Finally, 100 µl of each sample was loaded onto half-area black 96-well plates (#3686, Corning) and the fluorescence was read with excitation at 360 nm and emission at 460 nm at 25 °C on a Biotek Synergy HT microplate reader. Fluorescence signal of the negative control group was subtracted from each experimental sample. To analyse the desuccinylation activity of His-SIRT5, the same procedure was followed as for cell lysate, but instead of cell lysates, purified recombinant His-SIRT5 was used. The amount of His-SIRT5 used in each assay was indicated in figure legends.

### NAM sensitivity of desuccinylation activity

For analysing NAM sensitivity of cellular desuccinylation activity, the assay procedure was the same as desuccinylation assay described above, with a slightly different reaction system composition, as follows: 14 µl cell lysate (187.3 µg), 28 µl of 20 µM succinylated-substrate containing 1 mM NAD^+^, and 14 µl NAM (0.04–4000 µM, resulting in a final concentration in the reaction system of 0.01–1000 µM). The reaction was stopped with either 56 µl 1 × developer (experimental samples) or 56 µl 1 × developer containing 2 mM NAM (negative control sample). The fluorescence signal was subtracted from signal in the negative control group, which consisted of 14 µl cell lysate, 28 µl succinylated-substrate without NAD^+^ and 14 µl assay buffer. The same procedure was used to analyse NAM sensitivity of His-SIRT5 desuccinylation activity, except that His-SIRT5 (0.06 µg) was used instead of cell lysate.

### Deacetylation activity assay

Deacetylation activities of cell lysates and recombinant His-SIRT5 were determined similarly to the desuccinylation activity, except that an acetylated-fluorogenic substrate (#BML-KI179, Enzo) rather than a succinylated-substrate was used and 1 × developer II (#BML-KI176-1250, Enzo) was used to stop the deacetylation reaction.

### Western blotting

Cells were lysed in lysis buffer (50 mM Tris/HCl pH 7.4) containing deacylase inhibitors (1 µM trichostatin A and 10 mM NAM) as well as protease inhibitor cocktail (#04693159001, Roche) by sonication on ice, the cell lysate sonication procedure was the same as described in the Desuccinylation activity assays section. Samples were equalized to the same protein level with the lysis buffer following quantification by DC protein assays. Cell lysates (30 µg protein) were separated on NuPAGE 4–12% gradient gels (#NP0322, Thermo Scientific) at 110 V for 35 min, followed by 150 V for 40 min at room temperature. After which, proteins on the gel were immediately transferred to a nitrocellulose membrane at 300 mA for 1 h on ice. Antibodies against SIRT5 (#8782, Cell Signaling Technology), succinyllysine (#401, PTM Biolabs), voltage-dependent anion channel (VDAC) (#ab14734, Abcam) and Histone 3 (#9715, Cell Signaling Technology) were used to identify the respective targets. IRDye Donkey anti-rabbit (#926-32213, LI-COR Biosciences) and Goat anti-mouse (#926-32210, LI-COR Biosciences) were used and signals were detected using an Odyssey scanner (LI-COR). Lane profile analyses was performed using Image J 1.51p.

### Statistics

Statistical analysis was performed using GraphPad Prism 5.04. Data was compared using the two-tailed unpaired Student’s t-test. Data represent mean ± SEM. P values below 0.05 were considered significant.

## Supplementary information

Supplementary Figures.

## Data Availability

All data generated or analysed during this study are included in this published article (and its “Supplementary Information” files).
