# Supplementary material for: An optimized desuccinylase activity assay reveals a difference in desuccinylation activity between proliferative and differentiated cells
Source: Sci Rep. 2020 Oct 12;10:17030. doi: 10.1038/s41598-020-72833-7 (PMC7552388; doi:10.1038/s41598-020-72833-7)
Supplement: Supplementary file 1 — Supplementary Figures. [file 41598_2020_72833_MOESM1_ESM.pdf]

An optimized desuccinylase activity assay reveals a difference in desuccinylation activity between proliferative and differentiated cells

Taolin Yuan[1], Jaap Keijer[1], Angela H. Guo[2], David B. Lombard[2], Vincent C. J. de Boer[1,*]
[1]Human and Animal Physiology, Wageningen University & Research, Wageningen, 6708WD, the Netherlands. [2] Department of Pathology, University of Michigan, Ann Arbor MI 48109. USA.

*Correspondence

Vincent C.J. de Boer, PhD

Human and Animal Physiology

Wageningen University & Research

De Elst 1, 6708 WD Wageningen

The Netherlands

+31 317 484699

Vincent.deboer@wur.nl

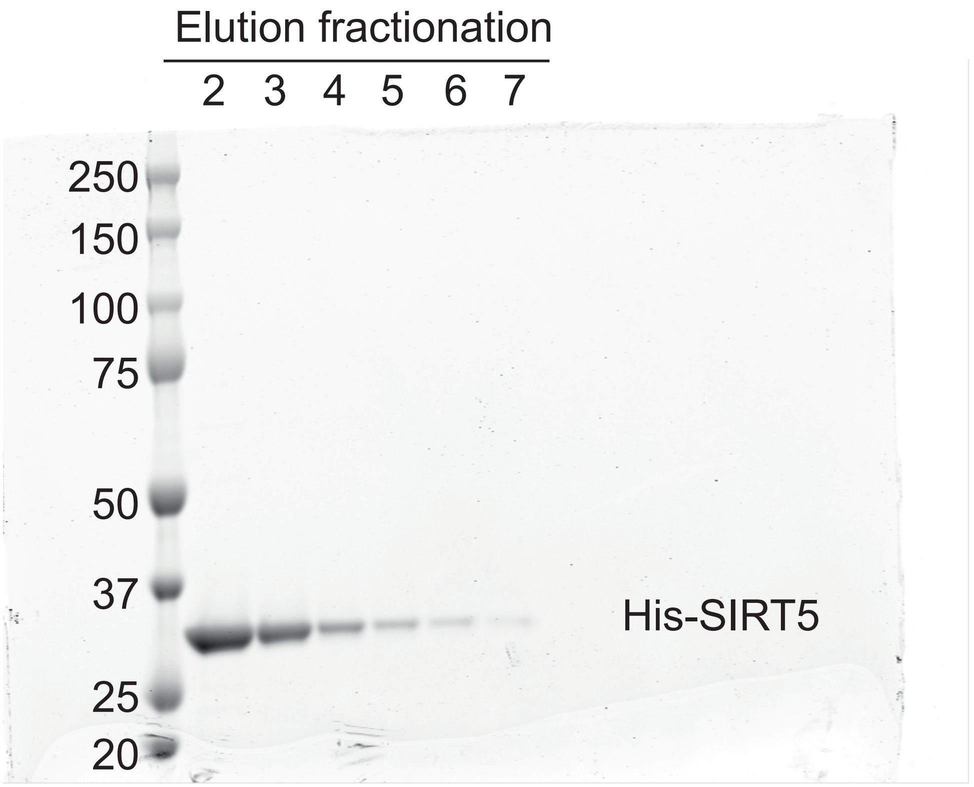

Supplementary Figure 1 Gel eletrophoresis result of elution fractionation of His-SIRT5. The elution fractionation 1 was not loaded on gel due to high salt content in the sample.

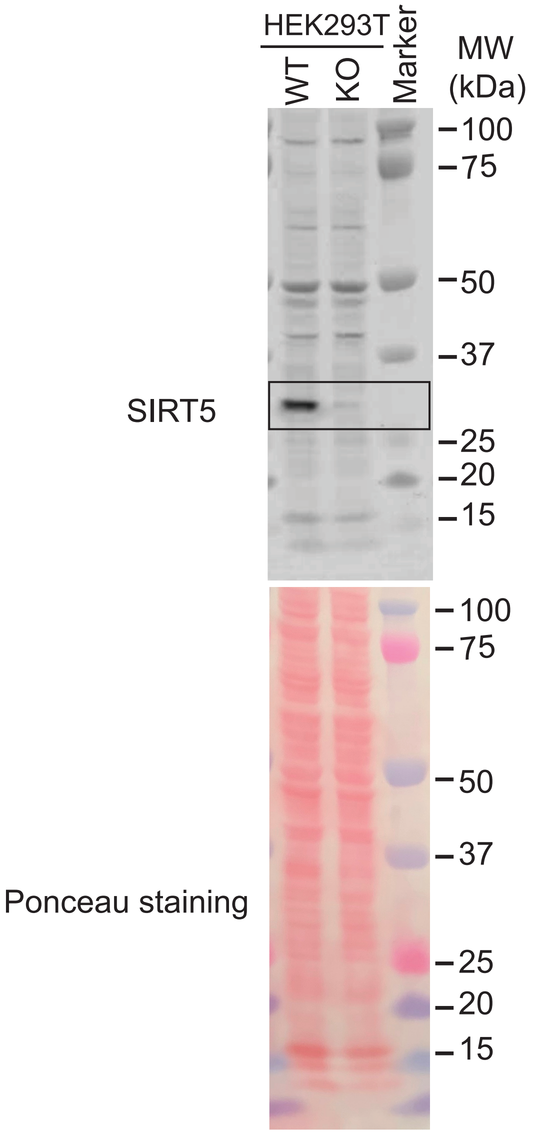

Supplementary Figure 2 Full-length blots for cropped blots shown in Fig. 1e. Ponceua staining shows the equal loading.

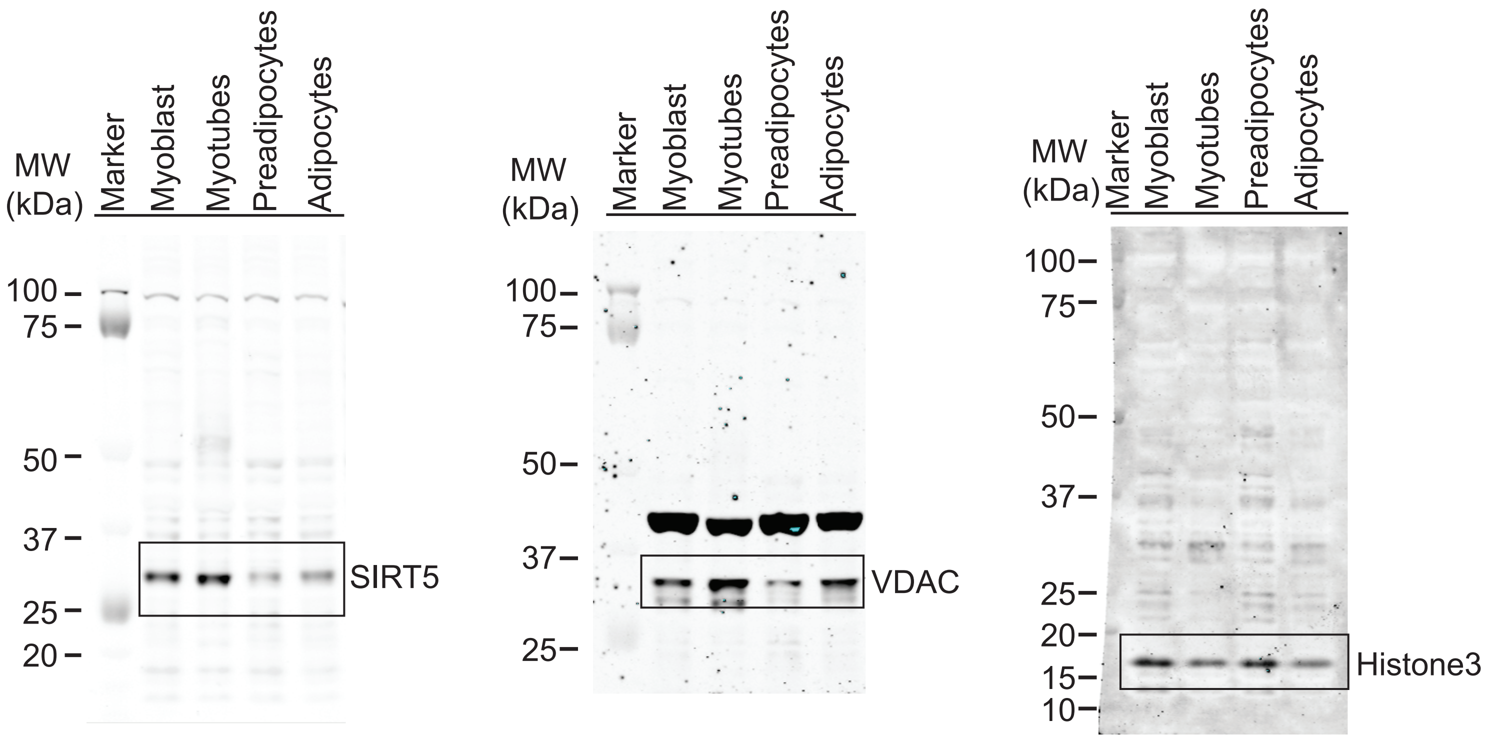

Supplementary Figure 3 Full-length blots for cropped blots shown in Fig. 5a.

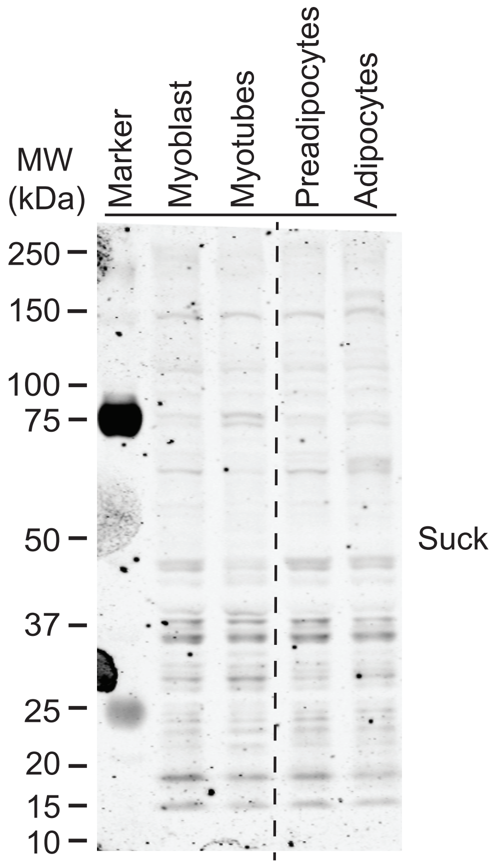

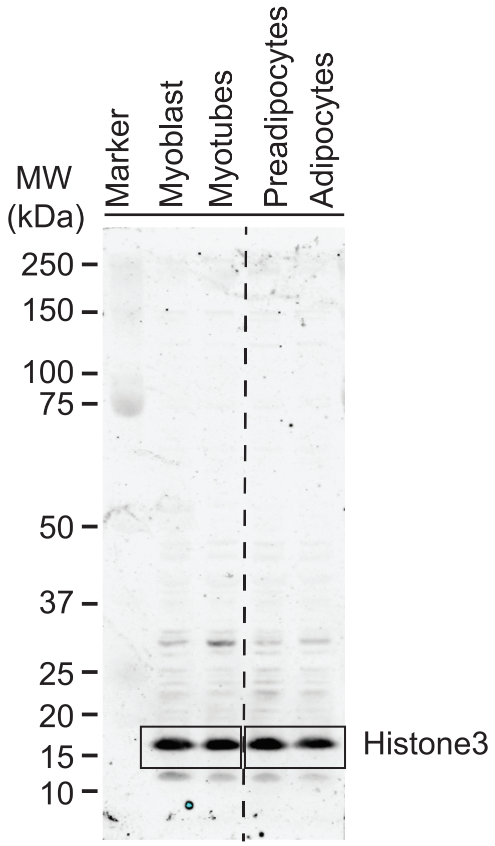

Supplementary Figure 4 Full-length blots of cropped blots shown in Fig. 5b and Fig. 5c. Blot was cropped along the black dash line. Left side of dash line was shown in Fig. 5b, and right side of dash line was shown in Fig. 5c.
